# Gene Expression in Granulosa Cells From Small Antral Follicles From Women With or Without Polycystic Ovaries

**DOI:** 10.1210/jc.2019-00780

**Published:** 2019-07-05

**Authors:** Lisa Ann Owens, Stine Gry Kristensen, Avi Lerner, Georgios Christopoulos, Stuart Lavery, Aylin C Hanyaloglu, Kate Hardy, Claus Yding Andersen, Stephen Franks

**Affiliations:** 1 Institute of Reproductive and Developmental Biology, Hammersmith Hospital, Imperial College London, London, United Kingdom; 2 Faculty of Health and Medical Sciences, Laboratory of Reproductive Biology, The Juliane Marie Centre for Women, Children and Reproduction, Copenhagen University Hospital, University of Copenhagen, Copenhagen, Denmark; 3 Wolfson Fertility Unit, Hammersmith Hospital, Imperial College Healthcare NHS Trust, London, United Kingdom

## Abstract

**Context:**

Polycystic ovary syndrome (PCOS) is the most common cause of anovulation. A key feature of PCOS is arrest of follicles at the small- to medium-sized antral stage.

**Objective and Design:**

To provide further insight into the mechanism of follicle arrest in PCOS, we profiled (i) gonadotropin receptors; (ii) characteristics of aberrant steroidogenesis; and (iii) expression of anti-Müllerian hormone (AMH) and its receptor in granulosa cells (GCs) from unstimulated, human small antral follicles (hSAFs) and from granulosa lutein cells (GLCs).

**Setting:**

GCs from hSAFs were collected at the time of cryopreservation of ovarian tissue for fertility preservation and GLCs collected during oocyte aspiration before *in vitro* fertilization/intracytoplasmic sperm injection.

**Participants:**

We collected hSAF GCs from 31 women (98 follicles): 10 with polycystic ovaries (PCO) and 21 without. GLCs were collected from 6 women with PCOS and 6 controls undergoing IVF.

**Main Outcome Measures:**

Expression of the following genes: *LHCGR, FSHR, AR, INSR, HSD3B2, CYP11A1, CYP19, STAR, AMH, AMHR2, FST, INHBA, INHBB* in GCs and GLCs were compared between women with PCO and controls.

**Results:**

GCs in hSAFs from women with PCO showed higher expression of *LHCGR* in a subset (20%) of follicles. Expression of *FSHR* (*P* < 0.05), *AR* (*P* < 0.05), and *CYP11A1* (*P* < 0.05) was lower, and expression of *CYP19A1* (*P* < 0.05), *STAR* (*P* < 0.05), *HSD3B2* (*P* = NS), and *INHBA* (*P* < 0.05) was higher in PCO GCs. Gene expression in GL cells differed between women with and without PCOS but also differed from that in GCs.

**Conclusions:**

Follicle arrest in PCO is characterized in GCs by differential regulation of key genes involved in follicle growth and function.

## Teaser

Follicle arrest in PCOS is characterized in GCs by differential regulation of genes involved in follicle growth and function: *LHCGR, FSHR, AR, CYP11A1, CYP19A1, STAR*, and *INHBA*.

Polycystic ovary syndrome (PCOS) is the most common endocrinopathy in young women, affecting up to 15% of women of childbearing age (1–4). Despite its prevalence, the underlying, complex pathophysiology of PCOS remains poorly understood. However, there is evidence for disordered ovarian follicular development that can be observed even at the early preantral stages (5). The later, gonadotropin-dependent, stages of follicle development are characterized by arrest at the small to medium-sized antral follicle stage and failure to progress to ovulation (5–7). The exact cause of aberrant follicle development is unknown, but follicle arrest is associated with elevated serum concentrations of LH, anti-Müllerian hormone (AMH), insulin, and androgens and a relative deficiency of FSH (8). Recent genome-wide association studies have found susceptibility loci close to genes encoding FSH receptor (*FSHR)*, FSH B polypeptide gene (*FSHB*), and LH choriogonadotropin receptor (*LHCGR)*, implicating gonadotropins and their receptors in the pathogenesis of PCOS (9–11).

Inappropriate (premature) responsiveness of granulosa cells (GCs) to LH may also play a part in follicle arrest in PCOS. Willis *et al.* (12, 13) examined GCs from human small antral follicles (hSAFs) taken from women with normal ovaries and regular cycles, which showed a response to FSH (as predicted) but no response to LH in terms of steroid production. GCs from women with anovulatory PCOS, however, showed significantly more variability of responsiveness to LH compared with ovulatory women [with or without polycystic ovaries (PCO)], with some follicles behaving as normal ovaries (*i.e.,* no response to LH), but with a significant subcohort displaying inappropriate responsiveness to LH. In addition to inappropriate responsiveness to LH, cultured GCs from hSAFs in PCOS women also show augmented estradiol and progesterone production in response to FSH (12, 13).

The aberrant LH responsiveness of GCs in PCOS is likely to be due to early acquisition of LHCGR (and/or their function) in GCs, which may result in terminal differentiation of GCs and subsequent arrest of follicle growth. However, Jeppesen *et al.* (14) detected *LHCGR* gene expression in GCs of follicles from healthy persons as small as 3 mm. Jakimiuk *et al.* (15) also studied GCs from women with PCOS and found increased gene expression of *LHCGR* and *CYP11A1* (catalyzing conversion of cholesterol to pregnenolone by side-chain cleavage) in GCs from hSAFs compared with cells from regularly cycling women. However, that study did not include quantitative RT-PCR analysis and has not been replicated, nor has a comprehensive study of expression of gonadotropin, androgen receptor, and steroidogenic enzymes been carried out.

Another factor implicated in the mechanism of arrested follicle growth in PCOS is AMH, a key member of the TGF-*β* superfamily of growth factors important for normal follicle development. Furthermore, researchers recently reported that treatment of pregnant mice with AMH results in a PCOS-like phenotype in the offspring, via a mechanism that implies a neuroendocrine action of AMH (16). Most of circulating AMH is produced by GCs of large preantral and small antral follicles (17, 18). AMH inhibits FSH-induced estradiol production by GCs *in vitro* (19) and, because AMH production is higher in women with PCOS (20, 21), the higher ovarian levels of AMH are thought to impair antral follicle function and contribute to follicle arrest (22). It has been concluded, on the basis of correlating serum AMH with antral follicle count on ultrasonography, that the production of AMH per follicle is increased in PCOS (23, 24). This view is supported by a study showing that concentrations of AMH (as measured by ELISA) were much higher in conditioned media of cultured GCs from women with PCOS compared with follicles from controls, although concentrations of AMH follicular fluid between polycystic and normal ovaries were not measured (25). Although *AMH* gene expression has been measured in luteinized GCs from gonadotropin-stimulated cycles (and reported to be higher than normal in women with PCOS) (26) it has not, to our knowledge, been measured in GCs from individual, matched, unstimulated follicles in women with and without PCO.

Other members of the TGF-*β* superfamily are plausible candidates for disrupted follicle function in PCOS. Expression of GDF9 and BMP15 has been found to be reduced in the oocytes of women with PCOS (27). Follistatin, produced predominantly in GCs (28), inhibits FSH secretion by binding and neutralizing activin action (29, 30), and has been shown to be elevated in serum of women with PCOS (31). Conversely, one study found that inhibins A and B, which also suppress FSH secretion, were lower in follicular fluid in large size-matched follicles from women with PCOS (32).

Androgens and insulin may also be important in the mechanism of follicle arrest in PCOS. Insulin can stimulate GC steroidogenesis and enhance GC responsiveness to LH (33), and increased androgen synthesis may augment FSH receptor signaling, both of which may increase local production of cyclic AMP and encourage terminal differentiation of GCs (8). However, it is unclear whether insulin and androgen receptors themselves are differentially expressed in PCOS GCs.

The principal objective of this study therefore was to use GCs from small antral follicles to investigate (i) the expression profile of receptors for the key regulatory hormones implicated in follicle arrest in PCOS, focusing on *LHCGR* and *FSHR*; (ii) the differences between PCO and control GCs in gonadotropin-responsive steroidogenesis *in vitro*; and (iii) the differential expression of TGF-*β* growth factors (particularly AMH) in hSAFs. In addition, we wished to compare gene expression profiles in GCs from hSAFs with those in GL cells from large, mature, antral follicles, which have frequently been used as a model of GC function in women with and without PCOS.

## Materials and Methods

### Study participants

GCs were collected from hSAFs collected at the time of cryopreservation of ovarian tissue for fertility preservation. The follicles were collected randomly and not timed to a particular point in the menstrual cycle. GCs were collected from 98 follicles from 31 women: 10 with PCO and 21 with normal ovaries. The ethical committee of the municipalities of Copenhagen and Frederiksberg approved the study (journal number; H-2-2011-044). Polycystic ovaries were defined primarily on the basis of ovarian volume (>10 mm^3^). Because these samples were taken from women with cancer who were about to undergo cancer treatment, data on menstrual history or hirsutism were not routinely recorded. However, biochemical indices of PCOS were measured and are reported in Table 1. Granulosa lutein cells (GLCs) were collected at the time of oocyte retrieval as part of *in vitro* fertilization or oocyte preservation in consenting women. Samples were collected from six women with PCOS and six women without. All women with PCO in this part of the study had oligomenorrhea or amenorrhea and therefore fulfilled the Rotterdam diagnostic criteria for PCOS. Hammersmith and Queen Charlotte’s Research Ethics Committee, London, United Kingdom, also approved the study (Reference 08/H0707/152). All participants provided informed consent.

**Table 1.** Patient Clinical Information (GC Samples From hSAFs)

Characteristic	Control (n = 49 Samples)	PCO (n = 49 Samples)	*P* Value
Age, y	25.7 ± 5.9	26.6 ± 5.5	0.67
Women, n	21	10	
Follicles included per woman, n	2.5 ± 1.5	4.9 ± 1.7	0.0004
Total follicles aspirated per woman (range), n	1–6	7–14	
Ovarian volume, mL	7 ± 2	14 ± 2.8	<0.0001
Serum LH, IU/L	5.9 ± 4.7	9.2 ± 6.8	0.10
Serum FSH, IU/L	5.4 ± 2.8	5.5 ± 2	0.94
Serum AMH, pmol/L	17.8 ± 10.8	42 ± 24.9	0.001
Serum testosterone, nmol/L	0.5 ± 0.49	0.7 ± 0.37	0.22
Follicle size, mm	5.8 ± 1.4	5.9 ± 1.5	0.51
Diagnosis, n			
Breast cancer	6	4	
Lymphoma	5	1	
Brain cancer	4		
Medulloblastoma	1		
Aplastic anemia	1		
Sickle cell anemia	1		
Ovarian cancer	1		
Colorectal cancer	1	1	
Diamond blackfan anemia	1		
Sarcoma		4	

Values expressed with a plus/minus sign are the mean ± SD.

### GC collection and isolation from small antral follicles

The fertility preservation procedure normally involved excision of one entire ovary. Individual, visible antral follicles were aspirated with a 23-gauge needle attached to a syringe. The diameter of the follicles was calculated by using a formula based on the aspirated volume (18). Follicular fluid was centrifuged at 1000 rpm for 3 to 5 minutes to isolate GCs. Larger numbers of peripheral follicles were noted in the PCO ovaries, and GCs were aspirated from 7 to 14 follicles from women with PCO compared with 1 to 6 follicles from normal ovaries. The supernatant was removed, and GCs were washed in PBS and centrifuged at 1000 rpm for 3 to 5 minutes. The PBS was discarded, and GCs were snap-frozen in liquid nitrogen. The samples were collected and stored at the Laboratory of Reproductive Biology, Rigshospitalet, Denmark.

### GLC collection, isolation, and culture

Follicular fluid was aspirated and pooled from follicles of individual women during the retrieval of oocytes for in vitro fertilization (IVF)/intracytoplasmic sperm injection. GL cells were extracted by centrifugation at 1000 rpm for 5 minutes to separate the fluid from cells, as previously described (34, 35). The cell pellets were resuspended in M199 (ThermoFisher Scientific, Waltham, MA) and layered onto a 45% Percoll (GE Healthcare, Chicago, IL) gradient and then centrifuged at 1600 rpm for 30 minutes to separate red blood cells. The GL cells at the interface were collected and washed with Dulbecco PBS (ThermoFisher Scientific). The cells were then cultured at a density of 1 × 10^5^ for 4 days in media (DMEM F-12 HAM with 10% FBS, ThermoFisher Scientific) to allow recovery from potential effects of exogenous hormones (34) and then cultured in serum-free media for 24 hours before being stored at −80°C or undergoing immediate extraction of RNA.

### Quantitative RT-PCR

RNA was extracted from cells using RNeasy® Plus Mini Kit (Qiagen Inc., Valencia, CA) according to manufacturer’s instructions. The RNA samples were stored at −80°C. RNA was reverse-transcribed into cDNA using the Invitrogen superscript IV First Strand synthesis system (Invitrogen, Carlsbad, CA). Quantitative, RT-PCR was carried out on 384 well plates using POWER SYBR Green (Applied Biosystems, Foster City, CA) according to the manufacturer’s instructions on an Applied Biosystems 7900 HT instrument. Primer sequences are listed elsewhere (36). Primer efficiency was calculated by using LinReg software (37); 90% to 110% was considered efficient. A melt curve was completed to ensure a single product was formed. The relative mRNA expression of each gene was calculated by using the housekeeping genes *β*-actin and *β*_2_-microglobulin, which have been validated as stable reference genes in these cells (38).

### Statistical analysis

Statistical analysis was completed by using Prism software, version 6 (GraphPad Inc,. San Francisco, CA). Unpaired *t* test and ANOVA were used to compare means of normally distributed data (patient data and serum results), and these data are expressed as mean ± SEM. Mann-Whitney and Kruskal-Wallis tests were used to compare the distribution of gene expression data, which were not normally distributed. These data are expressed as medians + 95% CIs. Correlations were completed by using a Spearman correlation. A *P* value < 0.05 was considered to indicate a statistically significant difference.

## Results

### Patient demographic characteristics, clinical information, and sample details

GCs were collected from unstimulated hSAFs from 31 women undergoing ovarian cryopreservation: 10 with PCO and 21 with normal ovaries and regular cycles. The women were aged 16 to 34 years, and the mean age was similar in women with and those without PCO (Table 1). Circulating AMH (*P* = 0.001), ovarian volume (*P* < 0.001), and number of follicles aspirated were higher in the women with PCO (Table 1). Their underlying cancer diagnoses are listed in Table 1.

**Figure 1.**
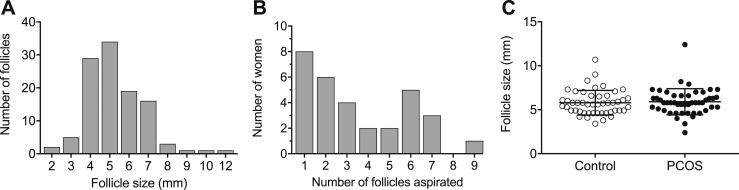
Number and size of follicles included in the study. (A) Number of follicles per follicle diameter (in millimeters). (B) Number of follicles included per woman. (C) Distribution of follicle sizes between control and PCO samples. The mean ± SD follicle size was 5.8 ± 1.4 mm for control samples and 5.9 ± 1.5 mm for PCO samples.

Ninety-eight samples were included: 49 from PCO women and 49 from controls. The diameter of the hSAFs from which the GCs were extracted ranged from 2.4 to 12.4 mm, and the mean (±SD) follicle size was 5.9 ± 1.4 mm (Fig. 1). The mean was similar for both control and PCO samples (Fig. 1). The number of follicles included per woman ranged from 1 to 9 (Fig. 1B). The mean (±SD) number of follicles included from each woman was 2.5 ± 1.5 from control women and 4.9 ± 1.7 from women with PCO.

GLCs were collected, during oocyte retrieval, from 12 women: 6 with PCOS and 6 without (controls) who were undergoing IVF/intracytoplasmic sperm injection or, in one case, oocyte cryopreservation (Table 2). Women with PCOS were younger (32 ± 1.4 vs 36 ± 3.8; *P* = 0.056), and received a lower dose of FSH (2075 ± 700 vs 3438 ± 1259; *P* = 0.06). There were no other differences between their IVF management protocols.

**Table 2.** Patient Clinical Information (GLC Samples)

Characteristic	Control (n = 6)	PCOS (n = 6)	*P* Value
Age, y	36 ± 3.8	32 ± 1.4	0.056
Indication for IVF, n	Unexplained: 5 Egg freezing: 1	Anovulatory PCOS	
FSH dose, IU	3438 ± 1259	2075 ± 700	0.06
Maturation trigger	hCG	hCG	
IVF protocol	Antagonist: 5 Flare: 1	Antagonist	

### Gonadotropin, androgen and insulin receptor expression in GCs and GLCs

**Figure 2.**
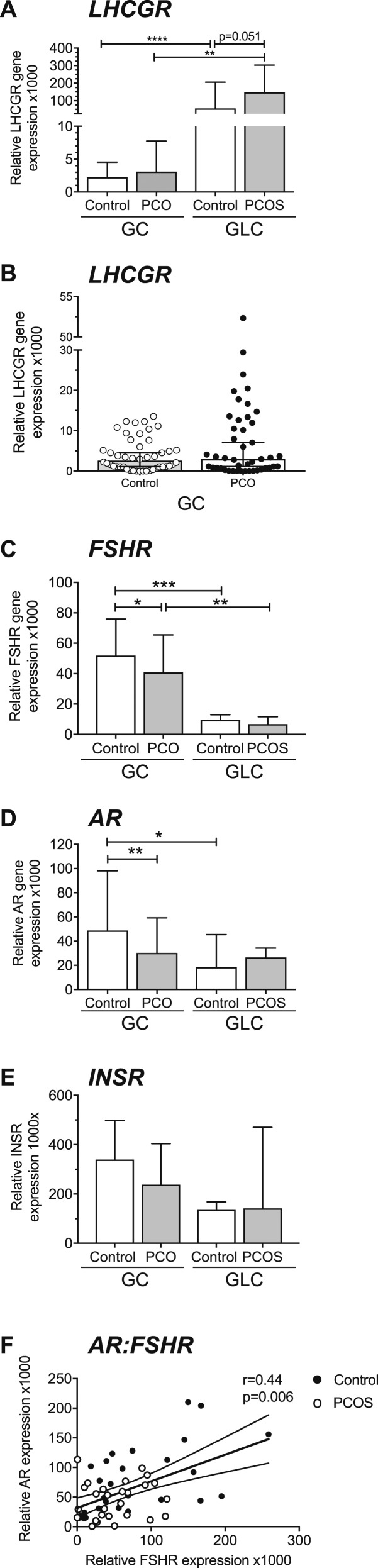
Gonadotropin, androgen, and insulin receptor expression (median + 95% CI). (A) *LHCGR* was higher in control and PCOS (GLCs) (n = 6) than in control and PCO GCs from small antral follicles (****P < 0.0001, **P < 0.01) (n = 49, n = 49). *LHCGR* was higher in PCOS GLCs than in controls (*P* = 0.051). *LHCGR* expression did not significantly differ between PCO and control GCs; however, in the PCOS group, a subset of samples (20%, 10 of 49) had an expression between 5 and 20 times higher than the mean expression of *LHCGR*. (B) Scatter plot of *LHCGR* in control and PCO GCs demonstrating individual PCO samples with higher *LHCGR*. (C) Median *FSHR* expression was higher in control and PCO GCs than in control and PCOS GLCs (***P < 0.001, **P < 0.01). *FSHR* expression was lower in PCO GCs than in controls (*P = 0.03). *FSHR* expression was the same in control and PCOS GLC. (D) *AR* expression was 3.5-fold lower in control GLCs than in GCs (*P < 0.05). *AR* in PCO GCs was half that of control samples (**P < 0.01). *AR* in PCOS GLCs was similar to that in control GLCs. (E) *INSR* expression was the same in control and PCO GCs and GLCs. (F) *AR* and *FSHR* expression were positively correlated (*P* = 0.006, r = 0.44).

*LHCG*R expression was detectable in all samples. Median *LHCGR* expression was 25 times higher in control GLCs than GCs (*P* < 0.0001) and was 48 times higher in PCOS GLCs than in PCO GCs (*P* < 0.01) (Fig. 2A). *LHCGR* was higher in PCOS GLCs than in control GLCs (*P* = 0.051) (Fig. 2A). Overall expression of *LHCGR* was low in GCs compared with GLCs. Although median values of *LHCGR* in GCs did not significantly differ between controls and women with PCO, the PCO group included a subset of follicles (n = 10; 20% of total follicles and from 70% of patients with PCOS) in which relative expression levels were above the normal range (Fig. 2B). These follicles showed expression levels between 5 and 20 times higher than the median expression of *LHCGR*. Expression in these 10 samples (1000× relative gene expression range, 13- to 52-fold) overlapped with and was not significantly different from that of GLCs from women without PCOS (range, 16- to 205-fold).

**Figure 3.**
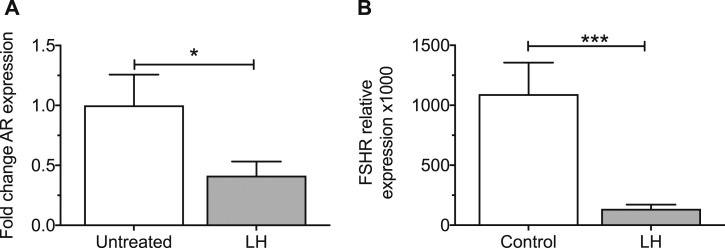
Androgen and FSH receptor expression are downregulated *in vitro* by LH. (A) *AR* gene expression in GLCs cultured with and without LH for 24 h (mean + SEM). LH reduced AR expression (*P < 0.05) (n = 8). (B) *FSHR* expression was reduced in granulosa lutein cells cultured with LH 10 nM for 24 h (mean + SEM) (***P < 0.001).

Overall, *FSHR* expression was higher in GCs from hSAFs than in GL cells, both in women with normal ovaries (*P* < 0.01) (Fig. 2C). *FSHR* gene expression was lower in GCs from women with PCO than in control samples (*P* < 0.01), whereas there was no significant difference between control and PCOS GLCs. Androgen receptor (*AR*) expression in control GCs was 3.5 times higher than in control GLCs (*P* < 0.05) (Fig. 2D) but did not differ between PCO GCs and PCOS GLCs. *AR* was lower in PCO compared with control GCs (*P* < 0.01) (Fig. 2C). Insulin receptor gene (*INSR)* expression was similar in GCs and GLCs and between controls and women with PCOS (Fig. 2E). There was no correlation between *LHCGR* and *AR* or *FSHR*. There was a strong correlation between *AR* and *FSHR* (*P* = 0.006) (Fig. 2F). Treatment of GLCs by LH *in vitro* significantly reduced expression of *AR* and *FSHR* (Fig. 3).

### Steroid enzyme gene expression

**Figure 4.**
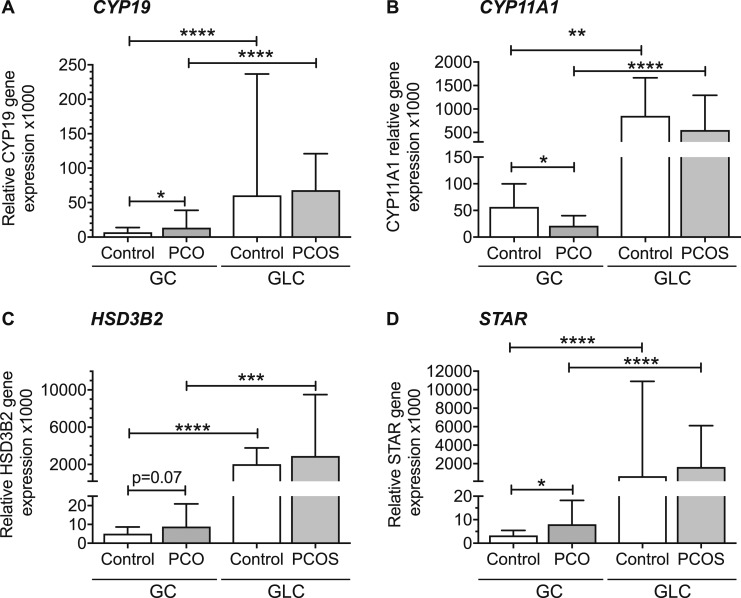
Gene expression of steroid enzymes in control and PCOS GCs and GLC (median + 95% CI). (A) *CYP19* (aromatase) expression is twofold higher in PCO than control GCs (*P < 0.05). *CYP19* expression in GCs was 9 times higher in control GLCs than in GCs (****P < 0.0001) and 5 times higher in PCOS GLCs than in PCO GCs (****P < 0.0001). (B) Median *CYP11A1* was twofold higher in control GCs compared with PCOS GCs (*P < 0.05) but was not different between control and PCOS GLCs. *CYP11A1* expression in control GLCs was 15 times in higher than in GCs (**P < 0.01) and 26 times higher in PCOS GLCs than in PCO GCs (****P < 0.0001). (C) *HSD3B2* expression in control GLCs was 400 times higher than in GLCs (****P < 0.0001) and was 327 times higher in PCOS GLCs than in PCO GCs (***P < 0.001). There was a non-significant trend toward higher *HSD3B2* in PCO than control GCs (*P* = 0.07) and *HSD3B2* expression was similar in PCOS and control GLCs. (D) *STAR* expression in control and PCOS GLCs was 194 and 203 times higher than in control and PCO GCs, respectively (****P < 0.0001). *STAR* expression was 2.5-fold higher in PCO GCs compared with controls (*P < 0.05).

Median aromatase (*CYP19*) gene expression in GCs was nine times higher in control GLCs than in GCs (*P* < 0.0001) and five times lower in PCOS GLCs than in PCO GCs (*P* < 0.0001). Median *CYP19* was twofold higher in PCO GCs than in controls (*P* < 0.05) (Fig. 4A). *CYP11A1* expression in control GLCs was 15 times higher than in control GCs (*P* < 0.01) and 26 times higher in PCOS GLCs than in PCO GCs (*P* < 0.0001) (Fig. 4B). Median *CYP11A1* was twofold higher in control GCs compared with PCOS GCs (*P* < 0.05). 3-*β*-hydroxysteroid dehydrogenase (*HSD3B2*) expression in control GLCs was 400 times in higher than in control GLCs (< 0.0001) and was 300 times higher in PCOS GLCs than in PCO GCs (*P* < 0.001) (Fig. 4C). There was a nonsignificant trend toward higher *HSD3B2* in PCO than control GCs (*P* = 0.069) and similar in PCOS and control GLCs. Steroidogenic acute regulatory protein *(STAR)* expression in control and PCOS GLCs was 194 and 203 times higher than in control and PCO GCs, respectively (*P* < 0.0001) (Fig. 4D). *STAR* was 2.5-fold higher in PCOS than in control GCs (*P* < 0.05). *STAR* and *HSD3B2* were both positively associated with LHCGR (*P* < 0.001) (data not shown).

### Growth factor gene expression

**Figure 5.**
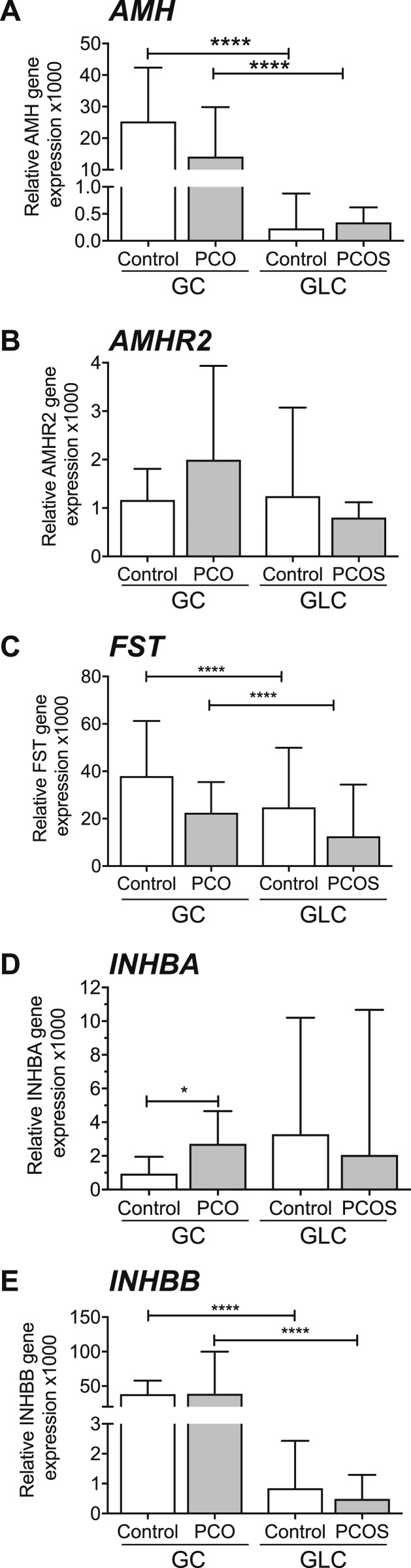
Gene expression of growth factors in GLC and GCs (median + 95% CI). (A) *AMH* expression did not differ between PCO and control GCs. *AMH* was negligible in GLCs (****P < 0.0001). (B) *AMHR2* expression did not differ between PCOS and control GCs. (C) *FST* (follistatin) expression did not differ between PCOS and control GCs and was higher in GCs than GLCs (****P < 0.0001, ****P < 0.0001). (D) *INHBA* was higher in PCO GCs compared with controls (*P < 0.05). (E) *INHBB* was higher in control and PCO GCs compared with control and PCOS GLCs (****P < 0.0001, ****P < 0.0001).

Whereas expression levels of *AMH*, follistatin (*FST*), and *INHBB* were all significantly higher in GCs than GLCs, they did not differ between PCO and control GCs (Fig. 5A, 5C, 5E). There was a correlation between *AMH* and *AMHR2* (*P* < 0.0001) (data not shown). *INHBA* but not *INHBB* expression was higher in PCO GCs than in control samples (*P* < 0.05) (Fig. 5D and 5E).

### Effect of follicle size on gene expression

To study whether differences between control and PCO GC samples depended on follicle size, we examined the impact of follicle size on expression of all genes studied. The distribution of gene expression in different follicle sizes did not differ between control and PCO samples (*i.e.,* over the range of sizes of SAFs from controls or women with PCO, follicle diameter had no significant effect on gene expression) (39).

## Discussion

This is a systematic comparison of gene expression, using quantitative PCR, in GCs of unstimulated hSAFs between normal and polycystic ovaries. We examined expression of key genes involved in follicle growth and steroidogenesis with a particular emphasis on factors that we, and others, have previously implicated in the mechanism of follicle arrest and anovulation in PCOS (12, 13). We found evidence of aberrant expression of gonadotropin receptors, *AR,* and steroidogenic enzymes. These findings were largely confirmed in GCs from preovulatory follicles obtained in connection with IVF treatment in women with PCOS and healthy women.

In GCs from unstimulated hSAFs, *LHCGR* was expressed at low levels in samples from follicles of all sizes, from both control and PCO tissue, replicating a previous study of gene expression in follicles of healthy women (14). Receptor expression did not correlate with follicle size over the range of hSAFs used in this study. There was no overall increase in *LHCGR* in PCO; however, a subpopulation of PCO follicles (20%) expressed *LHCGR* that was up to 20-fold higher than the mean expression in GCs from normal ovaries. The lower level of *LHCGR* expression (in stark contrast to that of *FSHR*) in GCs from hSAFs is consistent with the observation that, despite low levels of gene expression in normal follicles, LHCGR is functional only once the antral follicle has reached a diameter of around 8 to 10 mm (and that in the dominant follicle alone) during the second half of the follicular phase of a normal cycle. This is supported by the lack of LH-responsive steroid production in GCs from healthy women until the follicle reaches a diameter of about 10 mm (12, 40). In a previous study of GCs in hSAFs obtained from women with established PCOS, we found that a significant proportion of GCs in hSAFs showed a (premature) steroidogenic response to LH *in vitro,* which we interpreted as reflecting aberrant expression of functional LHCGR receptors in these follicles (12). It was therefore of particular interest that, in the current study, we showed that expression of *LHCGR* was increased in a subpopulation of hSAFs in women with PCO.

Although we cannot assume changes in gene expression equates to altered protein expression and function, *LHCGR* expression did strongly correlate with that of both *STAR* and *HSD3B2*, which were higher in PCO GCs, suggesting the expression of *LHCGR* relates to GC steroidogenic activity and especially production of progesterone and 17-OH-progesterone. The augmented expression of *STAR* and *HSD3B2* in PCO is consistent with findings in animal model studies; for example, GCs from antral follicles of sheep that were exposed prenatally to androgens also displayed increased expression of these enzymes (41). StAR is important in delivering cholesterol to the mitochondria as a substrate for CYP11A1, the first enzymatic step in steroidogenesis, but, interestingly, expression of *CYP11A1* was significantly lower in GCs of PCO follicles. CYP11A1 is the key enzyme in metabolism of cholesterol to pregnenolone (and thereafter HSD3B2 catalyzes conversion to progesterone) but it is not unusual for there to be a discordance between gene and protein expression and/or function.

The underlying cause of hyperresponsiveness to LH in PCOS is not known. Insulin augments not only basal production of estradiol and progesterone but also LH-stimulated steroid accumulation in GC cultures (33). Insulin receptor expression was not increased in PCO follicles in this study, but insulin induction of LHCGR in GCs may have a part to play in women with PCOS who are characteristically hyperinsulinemic. High circulating LH levels and elevated LH pulse amplitude and frequency are typical biochemical features of PCOS (42); they may also contribute to follicle premature LH responsiveness. Why this occurs in some but not all hSAFs also remains unclear. One previous study in GCs from hSAFs found a significantly higher expression of *LHCGR* in PCOS, and, interestingly, this was a feature of most follicles analyzed in that study (15). We also demonstrated a higher *LHCGR* expression in GCs from PCO hSAFs and PCOS GLCs, as has been shown previously (43, 44).

We found a significantly lower expression of *FSHR* in GCs from hSAFs in PCO compared with control. FSHR is critical for FSH-mediated follicle growth and development. The opposite might have been expected, as it has been shown in mouse and primate ovaries that androgens induce FSHR (45, 46). There is higher production of estradiol, in response to FSH, by PCOS GCs *in vitro* (12), but increased sensitivity to FSH may be possible despite lower levels of FSHR expression (and this accords with the finding of increased expression of *CYP19* in our study). In a study of rat ovaries (47) sustained follicle stimulation by LH decreases *FSHR* mRNA levels, FSHR signaling and inhibits FSH-induced follicular growth, so it is possible that inappropriate LHCGR activation inhibits *FSHR* expression. In addition, we showed (Fig. 3B) that LH treatment *in vitro* downregulates *FSHR*; therefore, reduced *FSHR* may result from premature LH/LHCGR activity.

Reduced *FSHR* expression is also related to the significantly lower *AR* expression in GCs from hSAFs in PCO. *AR* expression correlated strongly with *FSHR*, as we have shown previously (48, 49), suggesting a close interrelationship. We and others have shown that androgen treatment *in vitro* enhances *FSHR* expression, although the mechanism is unknown (46). It is unlikely that *AR* directly regulates *FSHR* because an androgen response element has not been identified on the *FSHR* gene promoter.

We have shown that AR expression is under the control of LH (Fig. 3A). LH treatment *in vitro* reduces AR expression. It is possible that the early responsiveness to LH seen in PCOS, probably as a result of some follicles expressing aberrantly high LHCGR and LH activity, inhibits *AR* expression, which, in turn, reduces *FSHR* expression. This combination of enhanced LHCGR activity and low *AR* and *FSHR* expression may underpin the follicular arrest seen in PCOS. The action of LH on AR may also explain why AR falls dramatically in mature follicles after the LH surge. This pattern of expression predisposes to terminal differentiation of the GCs or “premature luteinization.”

It is notable that we found no differences between PCO and controls in gene expression of *AMH* or its receptor. This suggests that the raised circulating AMH, consistently observed in PCOS, results from a higher number of antral follicles rather than increased production of AMH per follicle. Indeed, Jeppesen *et al.* (50) found a highly significant association between GC *AMH* mRNA expression and AMH protein level in the corresponding follicular fluid in a large number of individual antral follicles from normal human ovaries. They showed that *AMH* correlates with *AMHR2*, which we also found. Furthermore, *AMHR2* was, likewise, not significantly different between control and PCO GCs in our study.

Inhibins A and B are lower in follicular fluid of size-matched follicles in PCOS (32). Gene expression in GCs in our study showed the opposite, with higher *INHBA* in PCO and no significant difference in *INHBB*. This discordance has been seen previously in mature follicles (51) and may reflect the temporal differences between GC gene transcription and protein translation and storage in follicular fluid. Follistatin expression in GCs in this study was not different between PCO and controls, which matches the unchanged *FST* gene expression that we previously observed (32). The importance of increased *INHBB* expression in PCO GCs remains unclear.

This study also highlights GC dynamics and changes in expression between those from small antral follicles and large, luteinized follicles. GCs from large luteinized follicles have dramatically higher expression of genes involved in progesterone synthesis (*LHCGR, STAR, HSD3B2, CYP11A1*) and lower expression of genes involved in growth and estradiol synthesis (*FSHR, CYP19A1, AMH, AMHR2, FST*) when compared with GCs from hSAFs.

The major strength of this study is the use of large numbers of unstimulated hSAFs from ovarian material that is normally very difficult to obtain. These GCs have not been exposed to exogenous gonadotropins as part of an ovarian stimulation process. This is, to our knowledge, the largest and most comprehensive study of GCs from hSAFs in PCO and provides valuable insight into the pathogenesis of follicle arrest.

The samples from hSAFs were obtained from women with clear morphological evidence of polycystic ovaries, but an obvious limitation of the study is that we could not determine whether there were clinical features of polycystic ovary *syndrome*. Nevertheless, the clear differences we found in gene expression between normal and PCO GCs remain valid and, if anything, may underestimate differences between normal follicles and those from women with PCOS. Other potential limitations of these data are the relatively small range of follicle size and the lack of data from follicles >8 mm. However, it is the follicles that fall within this range that undergo arrest in women with PCOS and in which we have previously shown significant differences in gonadotropin responsiveness between healthy women and those with PCOS (12, 13). We found no obvious correlation between gene expression and follicle size, as has been shown in some previous studies. However, that is probably because most of our samples were from follicles between 4 and 7 mm, whereas previously reported changes with follicle size were most pronounced in comparison of results in small vs large antral follicles (48). Finally, in interpreting the results in GL cells, it is important to consider that stimulation protocols can differ between women with PCOS and that exposure to exogenous gonadotropins *in vivo* may affect gonadotropin responsiveness *in vitro*. However, we have previously shown that culture of GLs for 4 days before *in vitro* stimulation allows recovery of both LHCGR and FSHR responsiveness (34).

**Figure 6.**
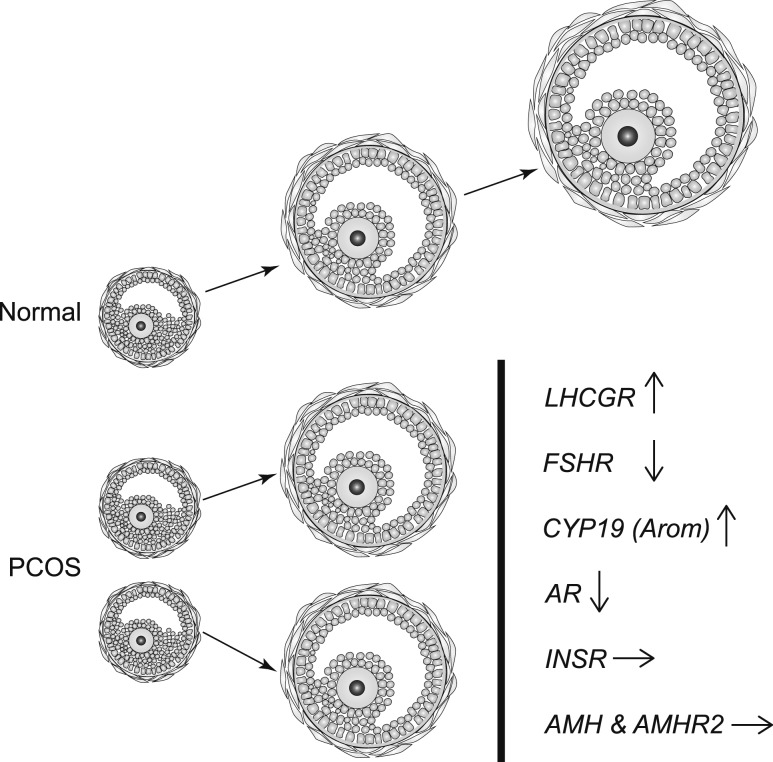
Summary of the key findings that we postulate are causally related to the mechanism of follicle arrest in PCOS. Expression of *LHCGR* is increased in a significant subpopulation of granulosa cells (GCs) in small antral follicles from PCOS. Increased LH activity may account for downregulation of both *FSHR* and *AR,* but increased aromatase (*CYP19*) expression is consistent with the notion that steroidogenesis is exaggerated, or at least conserved, in arrested follicles (8). Hyperinsulinemia may contribute to follicle arrest, but expression of insulin receptor (*INSR*) is unchanged. Expression of both *AMH* and *AMHR* is similar in normal and PCO follicles, and the role of AMH in arrested follicle growth in PCOS remains uncertain.

In conclusion, GCs from women with PCO display an altered profile of gene expression compared with women with regular cycles, with increased *LHCGR* in a subpopulation of follicles; reduced *CYP11A1*, *FSHR,* and *AR*; increased *STAR* and *CYP19*; and increased *INHBB*. There were, however, no differences between PCO and controls in *INHBA*, follistatin, *AMH,* or *AMH* receptor. We suggest that these changes (summarized in Fig. 6), in particular aberrant LHCGR function, but also reduced FSHR expression, are key factors leading to premature arrest of antral follicle growth in PCOS. This study contributes to the growing body of knowledge around the complex abnormal follicle dynamics in PCOS.

## Abbreviations:

AMH: anti-Müllerian hormone
GC: granulosa cell
GLC: granulosa lutein cell
hSAF: human small antral follicle
IVF: *in vitro* fertilization
PCO: polycystic ovary
PCOS: polycystic ovarian syndrome
